# Surgical versus non-surgical management of orbital fractures: study protocol for evidence generation of a prospective multicentre observational cohort registry

**DOI:** 10.1136/bmjopen-2025-100508

**Published:** 2025-07-30

**Authors:** Elske M Strabbing, The OFx Registry Study Group, Florian Thieringer, Eppo B Wolvius

**Affiliations:** 1 Erasmus MC University Medical Center, Rotterdam, The Netherlands

**Keywords:** ORTHOPAEDIC & TRAUMA SURGERY, Trauma management, Orthopaedic sports trauma, Fractures, Bone

## Abstract

**Introduction:**

There remains little consensus or guidelines for the clinical management of traumatic orbital fractures (OFx). The OFx Registry aims to increase real-world clinical evidence for the treatment of OFx via prospective, multicentre, international data collection. The primary objectives of this observational cohort study are (1) to document current treatment practices for and (2) to assess the outcomes of surgical and non-surgical treatment of orbital floor and/or medial wall fractures.

**Methods and analysis:**

Approximately 300 adult patients presenting with a displaced OFx in the orbital floor and/or medial wall will be enrolled prospectively over a recruitment period of ~36 months. All eligible patients treated either surgically or non-surgically as per routine standard of care will have follow-up assessments at 6 weeks, 3 months and 6 months post-treatment. Demographic data, injury details, treatment details and outcome measures will be documented in a cloud-based database. Outcome measures include clinical outcomes (eg, diplopia, extraocular motility, and condition of the eyelid, globe and soft tissues), radiological outcomes from collected images, patient-reported outcomes (eg, Diplopia Questionnaire and the newly developed AO Craniomaxillofacial (CMF) Injury Symptom Battery) and complications. A statistical analysis plan will be prepared before final analysis summarising the descriptive statistics to be used for data assessment. Appropriate research questions and statistical tests may be applied additionally, depending on the availability and quality of data collected.

**Ethics and dissemination:**

Ethics approval was obtained before patients were enrolled at each participating site. Patient enrolment followed an informed consent process approved by the responsible ethics committee. Peer-reviewed publications are planned to disseminate the study results.

**Trial registration number:**

NCT03887988.

**STRENGTHS AND LIMITATIONS OF THIS STUDY**

- The study protocol describes a prospective, international, multicentre registry collecting observational, real-world data from a large cohort of patients with fractures of the orbital floor and/or medial wall.
- By documenting routine standard of care of orbital fractures without intervention, the study will reflect real-world treatment patterns and clinical outcomes across diverse clinical settings.
- With a planned sample size of approximately 300 patients, the study will have sufficient statistical power for meaningful subgroup analysis, hypothesis generation and generalisability.
- Limitations of the observational study design include the lack of randomisation and lack of a control group, which limits the ability to establish causality or directly compare treatment effectiveness.
- Variability in treatment decisions across clinicians and geographical sites may lead to heterogeneity in the data and complex data interpretation but will potentially allow for extensive subanalyses and characterisation of related factors.

## Introduction

 Orbital fractures (OFx) in the craniomaxillofacial (CMF) region may occur after direct blunt trauma to the bony orbit, most frequently by motor vehicle accidents, sports injuries and assaults.^1^,^5^ OFx typically involves the thin bones of the orbital floor (ie, inferior wall) and/or medial wall.^4^ A fracture of the orbital floor and/or walls without a concomitant fracture of the orbital rim is called an orbital blowout fracture.^4^

Diagnosis of OFx via CT and cone beam CT (CBCT) is considered the gold standard method, with CT and CBCT imaging delivering detailed visualisation of the fractures at different angles.^4 6^ OFx can be managed surgically or non-surgically; however, currently, there is no consensus or guideline available on OFx treatment selection depending on defined factors, for example, precise indications for surgical versus non-surgical treatment, the optimal timing of surgery and the best surgical technique.^7^,^13^ Surgical treatment is indicated in cases of retrobulbar haematoma, muscle entrapment, persistent diplopia or clinically disturbing enophthalmos and/or hypoglobus.^14 15^ However, in numerous cases, the indication and timing of treatment is not straightforward because clinical symptoms, for example, duction deficits, and CT scan findings are inconclusive for decision making. In addition, there is conflicting evidence suggesting that surgical treatment may not always lead to better outcomes.^14^ Furthermore, delayed surgery or secondary reconstruction is sometimes necessary, particularly in cases of severe trauma, inadequate initial treatment or progressive anatomical disturbances.^16^,^19^ While surgical treatment aims to restore anatomy and function, it also carries inherent safety risks. Therefore, a decision for surgical treatment should not be made lightly.

These key questions regarding OFx management lack clear answers and characterisation. While recent studies have enhanced the availability of high-quality data and discussions on how patient demographics,^20^ surgical versus non-surgical management^21^ and the timing of surgery^12 22 23^ may impact clinical outcomes, results continue to remain inconsistent and inconclusive. Studies often lack standardised treatment protocols, are single centre, include populations of small sample size and/or demonstrate high heterogeneity in the cohort by failing to account for the diverse presentations of OFx. Furthermore, the long-term functional and aesthetic outcomes of different management strategies remain largely underexplored, limiting the ability to develop evidence-based guidelines.

This international, multicentre registry aims to document the current treatment practices for orbital floor and/or medial wall fractures, including primary treatment and secondary reconstruction, and their outcomes. By identifying and analysing current treatments of orbital floor and/or medial wall fractures, the study will highlight factors influencing clinical success and help determine optimal management strategies. The data collected will increase our knowledge about the treatment of these conditions and potentially may allow us to compare the clinical outcomes of surgical and non-surgical treatments. Additionally, the study aims to investigate the link between radiological parameters, clinical symptoms and treatment choice. The results may help formulate hypotheses, direct future clinical studies, provide better medical and clinical evidence and facilitate clinical decision making of OFx.

## Methods and analysis

### Study design and setting

This is a prospective, multicentre, international, observational cohort study serving as a registry for OFx treatment. Table 1 summarises the sites that are currently included in the study. All sites are academic tertiary care medical centres.

**Table 1:** Details of participating sites

Participating site	Country	Region
Universitätsspital Basel	Switzerland	Europe
University of Belgrade	Serbia	Europe
Hamad Medical Corporation	Qatar	Middle East
King Edward VIII Hospital	South Africa	Africa
Medizinische Hochschule Hannover	Germany	Europe
Rambam Health Care Campus	Israel	Middle East
12 de Octubre University Hospital	Spain	Europe
National Medical and Surgical Center named after N.I. Pirogov	Russia	Europe and Asia
Ludwig-Maximillians-Universität – Klinikum der Universität München	Germany	Europe
Federico II University of Naples Italy	Italy	Europe
Erasmus University Medical Center	Netherlands	Europe
University of California, Davis	USA	North America

### Objectives

Aside from collecting standard of care data into a large registry database and increasing our knowledge about the treatment and outcome of OFx, the specific objectives are (1) to document the current practice in both the primary and secondary treatment for orbital floor and/or medial wall fractures and (2) to describe and compare the outcomes of surgical and non-surgical treatment of orbital floor and/or medial wall fractures.

### Study design

This observational study protocol does not dictate any study-specific treatments, selection of materials or surgical techniques. All treatments will be performed according to the usual practice at participating sites and determined by the treating surgeons. Patients will be allocated to either the surgical or non-surgical group based primarily on local standard of care practices. This decision will be guided by the treating clinician’s judgement and relevant clinical indications, including individual patient characteristics and patient willingness to receive treatment. Post-treatment care and follow-up visits will also be conducted according to the standard procedures at participating sites.

Non-surgical treatments include either observation only, that is, no treatment or specific measures given, or conservative treatment with the use of supportive measures, for example, local antibiotics, anti-inflammatories, cold compresses and head elevation.

Primary surgical treatments will be classified as either ‘early’ or ‘delayed’ surgery when performed at ≤3 weeks or >3 weeks after the injury, respectively. Secondary reconstructions following unsuccessful primary surgical treatments of OFx will also be included in the study.

#### Inclusion criteria

- Age 18 years or older at the time of the injury.
- Patients with a displaced fracture of the orbital floor and/or medial orbital wall (areas 9, 10, 11, 15 and 16 according to the AO CMF Fracture Classification System,^24^ either diagnosed at the study site via CT or CBCT within 2 weeks of the injury or undergoing secondary reconstruction.
- Signed and dated Ethics Committee/Institutional Review Board (EC/IRB) approved written informed consent. For patients who are unable to provide independent written informed consent, written consent will be obtained according to the EC/IRB approved procedures.

#### Exclusion criteria

- Bilateral orbital fracture.
- Concomitant displaced fracture(s) of the orbital roof or any other area of the orbit.
- Concomitant ruptured globe.
- Displaced fracture of the malar bone.
- Displaced midface fracture.
- Displaced naso-orbito-ethmoid fracture or complex zygoma fractures.
- Presence at the time of enrolment of any disease with influence on eye motility (eg, strabismus, myasthenia gravis, Graves’ ophthalmopathy).
- Previous radiotherapy in the orbital region.
- Participation in any other medical device or medicinal product study within the previous month(s) that could influence the results of the present study.

#### Recruitment

The recruitment period will be ~36 months, during which approximately 300 patients are planned to be enrolled.

The participating sites will identify all consecutive eligible patients according to the defined inclusion and exclusion criteria. Eligibility assessment will be performed by the investigator and/or adequately trained member(s) of the research team. The assessment of eligibility includes but is not limited to an inquiry about the interest and willingness of the patient to participate in the research project. Data collection for this registry is not allowed without the consent of the patient or surrogate, and therefore, no data are collected in the electronic case report form (eCRF) prior to obtaining consent by the patient.

All consented patients will be allocated to a unique patient number. Each site will keep an Identification List linking the patient number with his/her personal information. Such Identification List is kept safe and in a locked place always. Sites are not allowed to share the Identification List with any third party except for the sponsor representative, legal authorities and EC/IRB, who may have access to the Identification List during monitoring/auditing activities performed on-site.

All consented patients should be followed up within the registry, except if their study participation is prematurely terminated.

#### Data collection

##### Baseline information

Demographic data (gender and year of birth) and medical history (eyelid disorders, globe asymmetry, visual field defects or blindness, and diplopia prior to the trauma) will be recorded for each patient.

##### Injury details

Injury details such the date of injury, mechanism, side of fracture, affected anatomical area, fracture characteristics (linear/defect), the dimensions of defect type fractures (maximum diameters in the anterior-posterior and lateral-medial directions) and the involved structures (nasolacrimal duct, lacrimal bone, internal orbital buttress (anterior and/or mid orbit), and the position and form of the rectus inferior muscle) will be recorded.

If a patient had primary reconstruction prior to enrolment, details on the primary implant and additional injury details such as the concurrent additional fractures, visual acuity, condition of the globe and its mobility, and the status of the eyelid will be documented as fully as possible.

##### Treatment details

For non-surgical treatment, the treatment date will be documented as the date of diagnosis.

Surgical details to be documented depending on whether it is primary or secondary reconstruction but may include the following as appropriate:

- Indications.
- Date of surgery.
- Time (in days) from trauma to surgery.
- Factors affecting timing of primary reconstruction, such as implant-related factors, hospital-related factors and clinical reasons.
- Surgery time (from skin incision to wound closure).
- Whether a forced duction test was performed.
- Surgical approach.
- Type of retractor used.
- Type(s) and material(s) of primary and secondary (if applicable) implant(s) used.
- Whether previous implants were removed.
- Use of computer-assisted methods for preoperative planning and/or intraoperative navigation.
- Use of antibiotics and steroids.
- Use of intraoperative imaging.
- Periorbital release, stripping and additional soft tissue procedures.

For patients who had primary reconstruction prior to enrolment, the primary treatment details will be documented as completely as possible.

##### Imaging data

Each site will take images and clinical pictures according to the local standard of care (routine) procedures. All images and pictures taken will be de-identified and sent to the sponsor.

##### Follow-up schedule

In general, patients will be followed up counting from Day 0 at approximately 6 weeks, 3 months and 6 months, as conforming to the routine visit schedule at the study site. These patients will remain active in the study for 6 months unless they received additional treatments.

The definition of ‘Day 0’ is different for different patients. For patients with acute fractures, if the patient:

- is treated non-surgically, Day 0 is the day of the diagnosis.
- is treated with primary ‘early’ or ‘delayed’ reconstruction, Day 0 is the day of the surgery.
- is treated with secondary reconstruction within the registry but had a primary reconstruction prior to enrolment, Day 0 is the day of the first surgery performed within this registry.
- is treated with primary reconstruction within the registry but then requires secondary reconstruction, Day 0 remains the day of the primary reconstruction and will not be shifted. The patients will be followed up at approximately 6 months after the secondary reconstruction according to the site’s routine visit schedule. These patients will remain active in the study for 6 months from the secondary reconstruction or up to the maximum of 1 year since Day 0.
- For patients needing more than one surgery within this study, they will remain active in the registry for 6 months after the last surgery but with the limit of maximally 1 year counting from Day 0.

The schedule for data collection at each visit is summarised in table 2.

**Table 2:** Schedule of follow-up visits and data collection

	Baseline	Treatment	Post-treatment visit 1	Post-treatment visit 2	Post-treatment visit 3	Additional treatment
	–	Day 0	6 weeks (+14 days)	3 months (+14 days)	6 months (+14 days)	Any time during follow-up
Eligibility	X					
Patient information/consent	X*					
Demographics and medical history	X					
Injury details	X					
Treatment details		X†				X
Clinical and functional outcomes	X‡		X	X	X	
Patient-reported outcomes	X§		X	X	X	
Adverse events/complications	X	X	X	X	X	X
Images (eg, CT, CBCT, X-rays)	X¶	X¶	X¶	X¶	X¶	X¶

* Informed consent may occur after treatment under certain circumstances (eg, treatment is immediate emergency, patient has temporarily impaired decision-making capacity, patient is unable to give written consent due to their injuries). No data can be collected in the eCRF prior to consent.

† For patients who undergo primary ‘delayed’ surgery, the Day 0 is considered as the day of the surgery.

‡ Clinical and functional outcomes at baseline refer to the status post injury and prior to treatment.

§ Patient-reported outcomes at baseline refer to the status post injury and prior to treatment.

¶ Only images (CT, CBCT, X-ray, etc) taken as part of the standard of care procedures will be collected. All images and pictures are de-identified.

CBCT, cone beam CT.

### Termination of participation

Patient participation in the study may terminate for reasons such as withdrawal of informed consent, ineligibility (screening failure), failure to commence treatment within this registry, loss to follow-up, death and termination of participation by the sponsor. Detailed information explaining the circumstances leading to the termination will be recorded in a dropout form. For these patients, the collected data will be censored as the day of termination and may be used in the analysis.

### Outcome measures

Clinical and functional outcomes, radiological outcomes and patient-reported outcomes (PROs) will be collected according to the schedule outlined in table 2.

#### Clinical and functional outcomes

The following will be documented:

- Condition of the eyelid and palpebral border.
- Condition of the globe such as its position, existence of anisocoria and/or strabismus and entrapment/impingement.
- Extraocular motility assessed by ‘follow my finger test’ in eight directions.
- Diplopia quantified by measuring the field of single binocular vision, ranging from 0 to 3:
  - 0: no diplopia.
  - 1: mild (diplopia appears more than 30 degrees from the primary position).
  - 2: moderate (diplopia appears between 10 and 30 degrees from the primary position).
  - 3: severe (diplopia appears within 10 degrees from the primary position).
- Vision: visual acuity will be measured using a visual test chart for both healthy and injured side with and without vision aids (as applicable) and expressed as percentage of visus.
- Sensory disturbances on the second branch of the fifth cranial nerve: anaesthesia, paraesthesia, hypoaesthesia or hyperaesthesia.

#### Radiological outcomes

The following will be measured centrally by one expert as described by Schouman *et al*^25^ using the collected images:

Pre-treatment parameters

- Degree of displacement of the fracture.
- Position of the inferior rectus muscle relative to the bone fragments.
- Shape of the inferior rectus muscle.
- Orbital volume of the injured compared with the uninjured side.
- Maximal height or periorbital tissue herniation.

Post-treatment parameters

- Orbital volume of the reconstructed compared with the uninjured side.

#### Patient-reported outcomes

The PROs used in this study are the Diplopia Questionnaire (DQ), the self-reported perception of visual acuity, strabismus and symmetry, and the AO CMF Injury Symptom Battery questionnaire.

DQ consists of seven questions concerning double vision in seven different gaze positions. It has been validated for responsiveness and test-retest reliability and has been shown to have good psychometric characteristics.^26^ The self-reported perception of visual acuity, strabismus and symmetry asks patients to evaluate on a 5-point Likert scale.

The AO CMF Injury Symptom Battery is the first PRO instrument developed specifically for traumatic craniomaxillofacial injuries with four modules specific to the injury site (oral, ocular, nasopharyngeal, ear) and five universal modules (pain/sensation, cognitive, cosmetic, psychosocial and injury impact).^27^ The items in this instrument were codeveloped by experts and patients and cognitive debriefing interviews were conducted with patients to assess the appropriateness and comprehensibility of the items.^27^ In the current registry, the ocular and psychosocial modules were used consisting of eye problems (7 items) and psychosocial problems (12 items). The questionnaire is available in English only and will be used only in English-speaking sites. The psychometric properties of this instrument have not been validated.

### Adverse Events

As this is an observational study, only anticipated condition-related, treatment-related or implant-related adverse events (AE) will be recorded from the time of consent onwards. These include AEs that are hardware-related (eg, malpositioning, extrusion, breakage or loosening of the implant), related to soft tissues (eg, infection, extraocular muscle entrapment, bleeding complications, persistent pain), related to the globe and vision (eg, globe dislocation, sclera show, epiphora, loss of vision), neurological-related (eg, mydriasis, optic nerve injury, infraorbital numbness, extraocular muscle palsy) and any other AE related to the OFx or OFx treatment.

Each AE will be followed up until resolved with or without persistent damage or until the end of the patient’s study participation, whichever occurs first. Unless it is indicated by the local EC/IRB, immediate reporting of AEs to the local EC/IRB is not required in this registry.

This registry can be considered as a minimal risk study given that participation does not pose additional risk as compared with the standard of care procedures.

### Statistical analysis

This is a registry-style observational study that aims to increase our knowledge about the treatment and outcome of OFx. Therefore, there is no formal statistical hypothesis and no formal sample size calculation. However, a detailed statistical analysis plan will be prepared before the final analysis documenting the descriptive statistics to be used for data assessment.

As the aim of the current registry is mainly descriptive, the patient characteristics, clinical data and outcomes recorded at routine standard of care scheduled follow-up assessments will be presented using simple summary statistics. Categorical variables will be summarised using the frequency and percentage for each category. Continuous variables will be summarised using the mean, SD, median, IQR, and minimum and maximum. Additionally, these summary statistics will be presented according to clinically relevant categories, for example, according to treatment received (conservative vs surgical treatment). Depending on the volume and the quality of the collected data, further appropriate statistics will be applied.

The AEs will be summarised both at patient level and AE level. AE rates with 95% CIs will be calculated based on the total population size, irrespective of dropouts during follow-up.

In general, all enrolled and eligible patients who were treated within the registry will be included in the analysis. If a patient discontinues participation due to any reason, all data collected before discontinuation will be integrated into the analysis. For specific research questions, only certain subgroups of patients may be relevant. In such a case, prior to the analysis, it will be defined which patients will be included in the analysis. Details concerning the handling of missing data and protocol violations will be specified in the statistical analysis plan.

### Data management

Data from participating patients are documented in eCRFs and captured in the REDCap Cloud Electronic Data Capture system (https://www.redcapcloud.com/). CRFs are to be completed in a timely manner and are password protected—only authorised personnel have access. After termination of the registry, each site will receive an electronic copy of its own data.

Images collected in association with this study will be de-identified and sent to the sponsor digitally.

Due to the observational nature of the study, a data monitoring safety board has not been implemented. Regular data monitoring and cleaning will be performed to ensure data accuracy.

### Current study status

Currently, 12 sites are participating in the study, with the majority being European sites. These sites are summarised in table 1. The first patient enrolment was in December 2019. The initial analysis of the study is expected to be completed by mid-2026.

## Discussion

Within the CMF community, there is a lack of consensus on OFx treatment strategy, including which symptoms and/or radiological parameters warrant a surgical versus non-surgical treatment approach. There is also a lack of consensus regarding the optimal surgical technique and best timing of the surgical intervention. This study protocol describes the collaborative efforts for evidence-based research on treatment management of orbital floor and/or medial wall fractures and factors which may influence clinical outcomes and safety measures.

The OFx Registry will prospectively collect multicentre data on a large, international, observational cohort within a real-world setting. The aim will be to detect meaningful, hypothesis-generating patterns in surgical versus non-surgical treatments and outcomes. Analyses of the OFX Registry data may help provide a valuable evaluation and documentation of OFx management to aid clinicians in implementing evidence-based protocols and minimising bias within standard of care. With a planned sample size of approximately 300 patients, the registry will offer sufficient statistical power for meaningful subgroup analysis, hypothesis generation and generalisability.

Limitations of the observational study design include the lack of randomisation and lack of a control group, which limits the ability to establish causality or directly compare treatment effectiveness. Additionally, given the multicentre participation in the study, there may be variance in data quality and/or different management trends observed between sites and between different regions, thus complicating the interpretation of outcomes. The strengths of multicentre data outweigh this limitation, however, as multicentre research enables a greater sample size and may provide greater clinical translation and application to a broader patient population across diverse clinical settings. If variability including clinician/geographical bias is detected during analyses, this may help to develop guidelines and a cost-benefit analysis to OFx management. Given the observational design, some bias of treatment strategy is expected and will be analysed and discussed in peer-reviewed publications stemming from the OFx Registry, for example, topics such as fracture severity based on recorded fracture details. Lastly, the absence of a standardised imaging protocol across sites due to the non-interventional design also poses challenges, as differences in imaging quality, timing and availability may affect data consistency.

This study protocol also describes how the OFx Registry may help to partially validate the AO CMF Injury Symptom Battery questionnaire as a newly established PRO. With the overarching aim within clinical care toward patient-centric, holistic approaches, it is critical for clinical research to measure PROs that impact recovery and daily quality of life, for example, occurrence and severity of pain and discomfort in daily tasks. Via a survey study between CMF surgeons and other sampled surgical fields, Joeris *et al* 2018 demonstrated that CMF surgeons are less familiar with PROs and use PROs less often as a study outcome, perhaps due to the lack of an established PRO specific to CMF traumatic injuries.^28^ The AO CMF Injury Symptom Battery questionnaire provides clinicians with a useful tool for assessing clinical outcomes from the patient perspective. Along with the need for psychometric validation, the limitation of the questionnaire is it is currently only available for English-speaking sites, and thus, results will have limited validity. Further studies with translated questionnaires in other languages will need to be performed to increase the validity of the tool. In the current study, only two of the nine modules described in the PRO were assessed, so further studies are required as well with all modules implemented. There was also no direct patient involvement for protocol development, so there may be measures important to patients that are missing from the registry. For the current study protocol, these limitations of the PROs will not have a significant impact on the quality of research outcomes.

This protocol defines the cooperative, multicentre effort of the OFx Registry Group to provide an open-ended, exploratory database for identifying OFx treatment trends and outcomes. The study will generate hypotheses for future research with the aim to standardise safe and effective OFx management of the highest clinical benefit.

## Ethics and dissemination

Ethics approval was obtained from the local ethics committee or institutional review board prior to patient enrolment.

The ethics committees and reference numbers (Ref. No.) for the OFx study are as follows: Ethikkommission Nordwest- und Zentralschweiz (Ref. No. 2019–01648); Ethic Committee of the School of Dental Medicine University of Belgrade (Ref. No. No 36/18); Institutional Review Board Hamad Medical Corporation Doha - Qatar (Ref. No. MRC-01-19-402); Biomedical Research Ethics Committee, University of Kwazulu-Natal (Ref. No. BE468/19); Rambam Medical Center Institutional Review Board (Ref. No. 0114–20-RMB); Comité de Ética de la Investigación con medicamentos del Hospital Universitario 12 de Octubre (Ref. No. 19/292); Local Ethical Committee of Federal State Budgetary Institution ‘National Medical & Surgical Center named after N.I. Pigrogov’, Ministry of Health of the Russian Federation (Ref. No. not applicable); Ethikkommission Medizinische Hochschule Hannover (Ref. No. 11454_BO_K_2024); Ethikkommission bei der LMU München (Ref. No. 19–580); Comitato Etico Universita Federico II (Ref. No. 245/20); Medische Ethische Toetsings Commissie Erasmus MC (Ref. No. MED-2019–0587); and UC Davis Institutional Review Board (Ref. No. 1470876).
