# Supplementary material for: Surgical versus non-surgical management of orbital fractures: study protocol for evidence generation of a prospective multicentre observational cohort registry
Source: BMJ Open. 2025 Jul 30;15(7):e100508. doi: 10.1136/bmjopen-2025-100508 (PMC12314983; doi:10.1136/bmjopen-2025-100508)
Supplement: Supplementary file 1 [file bmjopen-2025-100508.reviewer_comments.pdf]

# PEER REVIEW HISTORY

BMJ Open publishes all reviews undertaken for accepted manuscripts. Reviewers are asked to complete a checklist review form and are provided with free text boxes to elaborate on their assessment. These free text comments are reproduced below.

## ARTICLE DETAILS

### Title (Provisional)

Surgical versus nonsurgical management of orbital fractures: Study protocol for evidence generation of a prospective multicenter observational cohort registry

### Authors

Strabbing, Elske M; Study Group, OFx Registry; Wolvius, Eppo B

---

## VERSION 1 - REVIEW

---

| | |
|---|---|
| **Reviewer** | **1** |
| **Name** | **Holmes, Simon** |
| **Affiliation** | **QMUL, Department of Oral and Maxillofacial Surgery** |
| **Date** | **25-Mar-2025** |
| **COI** | **None** |

---

This is a research study that is badly needed within our profession and I applaud the effort. Of course the authors are respected peers and I also observe the attempt to get an international consensus. The authors should consider that the study will probably not empower the decision about when to operate ( like Burnstine did not achieve) but will probably demonstrate when conservative management yields less than perfect results - which can then be seen in context with the side effect profile. This study potentially could yield a real cost benefit analysis - I look forward to reading the outcomes.

---

| | |
|---|---|
| **Reviewer** | **2** |
| **Name** | **Nayak, Sunil** |
| **Affiliation** | **Manipal University, Oral and maxillofacial surgery** |
| **Date** | **30-Mar-2025** |
| **COI** | **None** |

---

The 'Methods and Analysis' needs to be modified. The 'severity of injury' has to be considered. Refer to comments in the pdf attached. Discussion needs to be modified accordingly. Poorly written 'Discussion'

****** The reviewer provided a marked copy with additional comments. Please contact the publisher for full details. ******

---

| | |
|---|---|
| **Reviewer** | **3** |
| **Name** | **Troise, Stefania** |
| **Affiliation** | **University of Naples Federico II** |
| **Date** | **18-May-2025** |
| **COI** | **None** |

---

The manuscript is well written, the topic is interesting and the data are well analyzed. The main problem of this work is that it is purely descriptive, so the goal is the description of the Authors' experience. In my opinion this should be better specified, even in the title. Another main problem is the bibliography: all the references are very old, there is no comparison with recent international scientific works, out of 27 references only 1 is from 2024, and 2 from 2018. I strongly advise the Authors to update the bibliography with recent epidemiological analyses: - Shen C, Shah JK, Nazerali R, Matthew MK. National Analysis of Surgical Intervention for Orbital Floor Fractures: Implications on Early Versus Late Repair. Ann Plast Surg. 2025 Apr 1;94(4S Suppl 2):S246-S252. doi: 10.1097/SAP.0000000000004270.

- Troise S, Committeri U, Barone S, Gentile D, Arena A, Salzano G, Bonavolontà P, Abbate V, Romano A, Dell'Aversana Orabona G, Vaira LA, De Riu G, Califano L, Piombino P. Epidemiological analysis of patients with isolated blowout fractures of orbital floor: Correlation between demographic characteristics and fracture area. J Craniomaxillofac Surg. 2024 Mar;52(3):334-339. doi: 10.1016/j.jcms.2023.10.007.

- Maunula MKK, Blomgren K, Sinkkonen ST, Nyman K, Tapiovaara LK. Isolated Orbital Fracture - Treatment and Outcomes in a Single Tertiary Care Centre. Craniomaxillofac Trauma Reconstr. 2024 Dec;17(4):NP163-NP171. doi: 10.1177/19433875241247370.

These are just a few examples of references that Authors could add to enrich the discussion and give a valid reason to publish their work, making it clear what is currently missing in the literature.

---

## VERSION 1 - AUTHOR RESPONSE

**REVIEWER #1**

**Dr. Simon Holmes, QMUL**

**Comments:**

**This is a research study that is badly needed within our profession and I applaud the effort. Of course the authors are respected peers and I also observe the attempt to get an**

| | |
|---|---|
| **international consensus. The authors should consider that the study will probably not empower the decision about when to operate ( like Burnstine did not achieve) but will probably demonstrate when conservative management yields less than perfect results - which can then be seen in context with the side effect profile. This study potentially could yield a real cost benefit analysis - I look forward to reading the outcomes.**<br><br>**Response:**<br>**Many thanks Dr. Holmes – we are looking forward to sharing the results with the community and exploring many sub-analyses including the cost benefit analysis. It should hopefully be a good start to a better documented consensus.** | |
| **REVIEWER #2**<br><br>**Dr. Sunil Nayak, Manipal University** | |
| **Comments:**<br>*****Please see attached document for further comments*****<br>**The 'Methods and Analysis' needs to be modified. The 'severity of injury' has to be considered. Refer to comments in the pdf attached. Discussion needs to be modified accordingly. Poorly written 'Discussion'**<br><br>**Response:**<br>**Thank you Dr. Nayak for your thorough evaluation. Your advice on how to better communicate these sections is very much appreciated. You have brought up some difficult points of the study and lack of clear consensus in the community that we have tried to better emphasize. These points on e.g. severity, surgeon bias, and factors that may affect clinical outcomes are not clear yet in the literature; however, these points will all be thoroughly discussed and analyzed in future OFx publications once the clinical data has been collected. We look forward to sharing the initial results with the community to promote further discussions.** | |
| **2.1** | **Q:** Kindly clarify as to how will you allot patients into the surgical or nonsurgical treatment group.<br><br>There may be some who may not opt for surgery. |
| | **R:**<br>As per the study protocol of this non-interventional study, patients will be allocated to either the surgical or non-surgical group based primarily on standard of care practices. This decision will be guided by the treating clinician's judgment and relevant clinical indications, including individual patient characteristics. As per usual standard of care, the patient's interest and willingness to undergo treatment will also be important. We have now added a statement to the methods section of the revised manuscript for better clarity. Please also see the response to 2.3 for more clarity. |
| **2.2** | **Q:** All patients with displaced fractures may not be ideal candidates for non surgical treatment . Are you following any particular criteria to group them into non-surgical group? |

| | |
|---|---|
| | **R:** Please refer to the above response to 2.1. |
| **2.3** | **Q:** Are you classifying patients based on severity of orbital trauma? If the results are analysed without taking into consideration the severity of injury, there is bound to be a huge bias. Kindly clarify |
| | **R:**<br>We agree that classifying the fractures based on severity is important to carry out unbiased comparison. Study data collection includes fracture details that can encompass varying clinical, anatomical, and contextual factors indirectly reflecting fracture severity.<br>When analyses are performed, we will explore this factor and how it may be affecting clinical outcomes and measures of safety. Especially at the start of the study in December 2019, surgeons typically did not apply any consistent or specific severity index for orbital blowout fractures during standard of care. The exploratory nature of this current registry study is to generate potential analyses on treatment decisions and factors that may affect outcomes, which may also vary between sites and surgeons. Thus, some inter-/intra-variability and bias between clinicians and sites may be expected for an observational study, but it will be discussed once the clinical data is assessed in future OFx publications. By documenting multi-site standard care without intervention, the study will reflect real-world treatment patterns across diverse clinical settings.<br>We have added a brief statement on this now in the discussion of the revised manuscript to provide better clarity. |
| **2.4** | **Q:** Kindly clarify on standard of care treatment for non-surgical group |
| | **R:** The information on treatment per group is located in the “Study procedures” section of the manuscript, now renamed “Study design” for use of more common wording. To add clarity on the current point, the treatment details have been slightly rewritten in the revised manuscript as follows:<br><br>Nonsurgical treatments include either observation only, i.e. no treatment or specific measures given, or conservative treatment with the use of supportive measures, e.g. local antibiotics, anti-inflammatories, cold compresses, and head elevation.<br><br>Primary surgical treatments will be classified as either “early” or “delayed” surgery when performed at ≤ 3 weeks or > 3 weeks after the injury, respectively. Secondary reconstructions following unsuccessful primary surgical treatments of OFx will also be included in the study. |
| **2.5** | **Q:** Discussion needs to be modified accordingly. Poorly written 'Discussion' |
| | **R:**<br>Thank you for suggesting the discussion should be improved. We admit it is difficult to write a discussion that is strictly based on the methods of a study, without getting ahead of ourselves on potential aims and outcomes. With the format and purpose of a protocol publication, the authors believe it is not suitable to discuss “normal” discussion topics, e.g. in-depth analysis of related clinical data in the literature, before the data of the current study is finalized. Many protocol publications do not have a |

| | |
|---|---|
| | discussion section; however, we believed it to be important to explain what we already know to be strengths and limitations of the study protocol. Once the OFx clinical data has been collected, findings from this study will then be thoroughly discussed and analyzed in the discussions of future OFx publications together with the most up-to-date literature and clinical data.<br><br>We have revised the discussion now of the current manuscript with clearer points on the strengths and limitations of the study methods (please also see the separate "Strengths and Limitations" box within the BMJ Open format and the full discussion in the manuscript, as we have just copied some examples below).<br><br>Added as strengths:<br>"With a planned sample size of approximately 300 patients, the registry will offer sufficient statistical power for meaningful subgroup analysis, hypothesis generation, and generalizability. By documenting standard care without intervention, the study reflects real-world treatment patterns across diverse clinical settings. Additionally, as an open-ended, exploratory registry, it provides a valuable platform for identifying treatment trends and generating hypotheses for future research."<br>Added as limitations:<br>"The lack of randomization or a control group limits the ability to establish causality or directly compare treatment effectiveness. Additionally, variability in treatment decisions across clinicians may lead to heterogeneity in the data, complicating the interpretation of outcomes. The absence of a standardized imaging protocol also poses challenges, as differences in imaging quality, timing, and availability may affect data consistency." |
| **REVIEWER #3**<br><br>**Dr. Stefania Troise, University of Naples Federico II** | |
| **Comments:**<br>**The manuscript is well written, the topic is interesting and the data are well analyzed.**<br><br>**Response:**<br>**Thank you very much Dr Troise for this positive feedback and enthusiasm on the OFx topic. You are very correct that a lot of valuable information on orbital fractures has been published in the last years, and we hope we could update the introduction suitably within the confines of the protocol publication format. When the initial publications are written on the clinical data acquired, we will also absolutely perform a complete updated systematic literature review of the current literature at that time to apply to both the introduction and discussion.** | |
| **3.1** | **Q:** The main problem of this work is that it is purely descriptive, so the goal is the description of the Authors' experience. In my opinion this should be better specified, even in the title. |

| | |
|---|---|
| | **R:** We agree that the protocol manuscript is purely descriptive and recognize that it may not be the most "exciting" value yet without the clinical data. Us authors would be very excited to have the study protocol published while the study is ongoing so the whole CMF surgeon community is aware of all the data and outcomes being collected. It is critical that hypothesis-generating discussions can already start to develop within the field, especially with such a large amount of potential data and potential sub-analyses in this study. This would produce the most valuable output once the data collection is finalized.<br>Regarding the manuscript title, to better emphasize this is a protocol publication specifically on the study methods, we would suggest a simple change of wording to the following:<br>**Surgical versus nonsurgical management of orbital fractures: Study protocol for evidence generation ~~through~~ ~~of~~ of a prospective multicenter observational cohort registry** |
| **3.2** | **Q:** Another main problem is the bibliography: all the references are very old, there is no comparison with recent international scientific works, out of 27 references only 1 is from 2024, and 2 from 2018. I strongly advise the Authors to update the bibliography with recent epidemiological analyses: - Shen C, Shah JK, Nazerali R, Matthew MK. National Analysis of Surgical Intervention for Orbital Floor Fractures: Implications on Early Versus Late Repair. Ann Plast Surg. 2025 Apr 1;94(4S Suppl 2):S246-S252. doi: 10.1097/SAP.0000000000004270.<br>- Troise S, Committeri U, Barone S, Gentile D, Arena A, Salzano G, Bonavolontà P, Abbate V, Romano A, Dell'Aversana Orabona G, Vaira LA, De Riu G, Califano L, Piombino P. Epidemiological analysis of patients with isolated blowout fractures of orbital floor: Correlation between demographic characteristics and fracture area. J Craniomaxillofac Surg. 2024 Mar;52(3):334-339. doi: 10.1016/j.jcms.2023.10.007.<br>- Maunula MKK, Blomgren K, Sinkkonen ST, Nyman K, Tapiovaara LK. Isolated Orbital Fracture - Treatment and Outcomes in a Single Tertiary Care Centre. Craniomaxillofac Trauma Reconstr. 2024 Dec;17(4):NP163-NP171. doi: 10.1177/19433875241247370.<br><br>These are just a few examples of references that Authors could add to enrich the discussion and give a valid reason to publish their work, making it clear what is currently missing in the literature. |
| | **R:** Thank you very much for raising this important point of providing key publications from recent years to be included in the manuscript. We have now updated the introduction and discussion within the confines of the format of a protocol, methods-based manuscript. We hope it is clear what is currently missing in the literature on OFx management and how this hypothesis-generating work could greatly help form a better written consensus on OFx treatment. Once the OFx clinical data has been collected, findings from this study will be thoroughly discussed and analyzed in future OFx publications, together with the most up-to-date and relevant literature discussed in both the introduction and discussion. |
